# Integration of Motion Responses Underlying Directional Motion Anisotropy in Human Early Visual Cortical Areas

**DOI:** 10.1371/journal.pone.0067468

**Published:** 2013-06-28

**Authors:** Wouter Schellekens, Richard J. A. Van Wezel, Natalia Petridou, Nick F. Ramsey, Mathijs Raemaekers

**Affiliations:** 1 Department of Neurology & Neurosurgery, UMC Utrecht, Utrecht, The Netherlands; 2 Rudolf Magnus Institute, UMC Utrecht, Utrecht University, Utrecht, The Netherlands; 3 Department of Biophysics, Donders Institute for Brain, Cognition and Behaviour, Radboud University Nijmegen, Nijmegen, The Netherlands; 4 Biomedical Signals and Systems, MIRA, University of Twente, Enschede, The Netherlands; 5 Radiology, UMC Utrecht, Utrecht, The Netherlands; University of Minnesota, United States of America

## Abstract

Recent imaging studies have reported directional motion biases in human visual cortex when perceiving moving random dot patterns. It has been hypothesized that these biases occur as a result of the integration of motion detector activation along the path of motion in visual cortex. In this study we investigate the nature of such motion integration with functional MRI (fMRI) using different motion stimuli. Three types of moving random dot stimuli were presented, showing either coherent motion, motion with spatial decorrelations or motion with temporal decorrelations. The results from the coherent motion stimulus reproduced the centripetal and centrifugal directional motion biases in V1, V2 and V3 as previously reported. The temporally decorrelated motion stimulus resulted in both centripetal and centrifugal biases similar to coherent motion. In contrast, the spatially decorrelated motion stimulus resulted in small directional motion biases that were only present in parts of visual cortex coding for higher eccentricities of the visual field. In combination with previous results, these findings indicate that biased motion responses in early visual cortical areas most likely depend on the spatial integration of a simultaneously activated motion detector chain.

## Introduction

Recently, several imaging studies have provided evidence for anisotropies in cortical responses to motion [1]–[4]. However, the cause of motion anisotropy, or directional motion bias, is not well understood. Raemaekers et al. [4] reported strong centripetal and centrifugal directional motion biases in BOLD responses compared to tangential motion directions in V1, V2, and V3. Importantly, the latter study reported that the directional motion biases disappeared, when motion was occluded by bars orthogonal to the path of motion, whereas the biases remained present when these occluding bars were positioned parallel to the path of motion. This finding is an important indicator that the directional motion biases, as reported by Raemaekers et al. [4], are related to the integration of motion responses across several motion detectors in visual cortex, instead of being the result of local inhomogeneities in the density of motion detectors tuned for a particular motion direction (a local-field inhomogeneity would produce directional motion biases regardless of the position of occluders). This indicates that directional motion biases emerge on a relatively large scale in retinotopic cortex [1], [5]. In addition, it suggests that directional motion biases are caused by an integration of motion information along the path of motion similarly as described in human psychophysical studies on motion recruitment [6]–[10]. An integration of motion information along the path of motion, implies a mechanism where aligned motion detectors influence neuronal activity of neighboring detectors when signaling a particular motion direction, thereby producing directional motion biases. Similar mechanisms have also been previously described in macaque physiological studies on visual neurons in extra-striate cortex, where multiple radially aligned neurons were necessary for the emergence of motion biases [11], [12].

**Figure 1:**
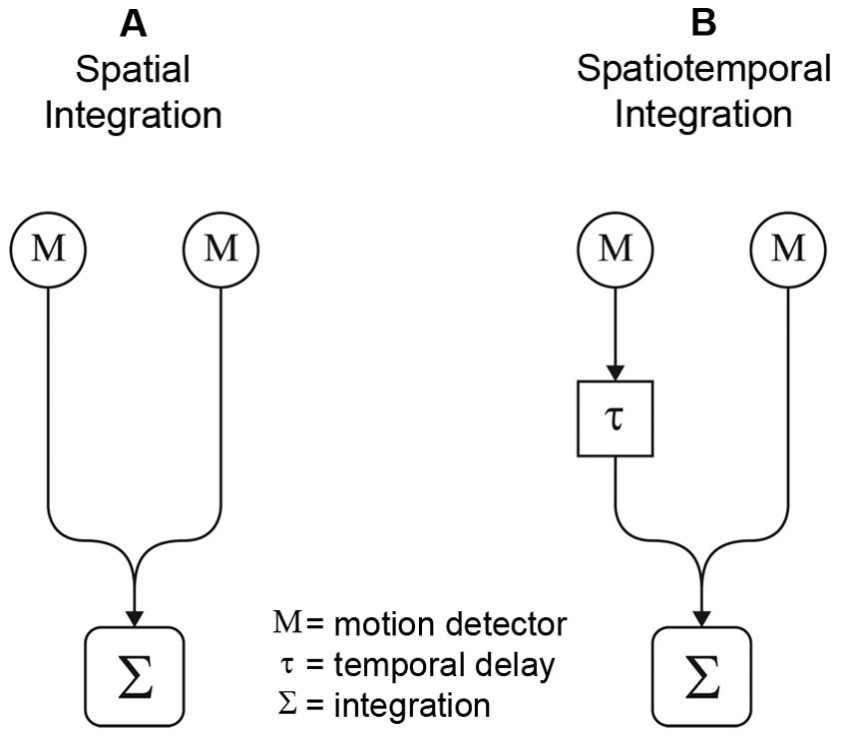
Simplified schematic of motion integration. Spatial integration (A) only includes spatial information from activated motion detectors, whereas spatiotemporal integration (B) also includes the temporal component of motion detector activity. This figure only displays integration over 2 motion detectors, aligned with the path of motion. The actual motion integration may well extend beyond 2 motion detectors.

The integration of aligned motion detector information can have two distinguishable characteristics that are tested in the current experiment. One option is that integration is only spatial [13]–[15], meaning that directional biases are dependent on the length of a chain of activated motion detectors. In that case, the length of a motion stimulus parallel to the path of motion determines the extent of the motion integration. A simplified schematic of spatial integration over just two motion detectors is presented in figure 1A. Alternatively, when the length of the motion stimulus is increased, not only the length of the activated detector chain increases, but also the duration that the individual dots are on the screen. It could, thus, be argued that interrupting the motion stimulus nullifies directional motion biases by interrupting the trajectories of the individual dots instead of interrupting an activated motion detector chain. A mechanism that keeps track of motion signals from individual dots along a motion trajectory would require a spatiotemporal instead of a spatial integration across aligned motion detectors [16], [17]. Therefore, if motion anisotropies are dependent on spatiotemporal information instead of only spatial information, then the duration of individual dots on screen is important for the emergence of directional motion biases. Spatiotemporal integration of motion responses would incorporate a temporal difference (delay) in activation of multiple spatially aligned motion detectors (figure 1B).

The following experiments are conducted to establish the nature of the integration of motion responses underlying directional biases in retinotopic cortex. We hypothesize that directional motion anisotropies emerge as a result of either spatial or spatiotemporal integration of activity of motion detectors. To discriminate these types of integration in early visual cortical areas, the spatiotemporal correlation of moving dots was disrupted (de-correlated) at fixed points in space (spatial decorrelation) and time (temporal decorrelation). By shortening the spatial extent of coherent motion along the path of motion through spatial decorrelations, motion information integration is limited to the area between spatial decorrelations. This would not discriminate between spatial and spatiotemporal integration of motion. However, if the duration of coherent motion is shortened, while the spatial extent of coherent motion covers more extensive portions of the visual field (temporal decorrelation), spatiotemporal integration will be affected, whereas spatial integration of motion information will not.

## Methods

### Subjects

Eleven subjects (mean age  = 24 years, 6 female) were recruited from Utrecht University. All subjects gave written informed consent for participation. The protocol was approved by the local ethics committee of the University Medical Center Utrecht, in accordance with the Declaration of Helsinki (2008).

### Scanning Protocol

Scanning was performed on a 7 Tesla Philips Achieva scanner (Philips Healthcare, Best, Netherlands) with a 16-channel receive headcoil (Nova Medical, MA, USA). Functional MRI (fMRI) measurements were obtained using an EPI-sequence with the following parameters: SENSE factor = 2.2, TR = 1500 ms, TE  = 25 ms, flip angle  = 80°, coronal orientation, FOV (AP, FH, LR)  = 52×169×169 mm^3^. The acquired matrix had the following dimensions: 26×96×96, voxel size: 2×1.75×1.75 mm^3^. The functional images were acquired from the posterior 52 mm of the brain, covering the occipital lobe, and were angulated along the z-axis to obtain an orthogonal orientation relative to the calcarine sulcus. Additionally, a T1-weighted image of the whole brain (0.49×0.49×0.50 mm^3^, FOV  = 512×380×512) and a proton density image of equal dimensions were acquired at the end of the experimental sessions.

### Stimuli

For stimulus presentation a desktop PC, a projector and a rear projection screen were used. The stimuli were programmed using C++ software (Stroustrup, 1983, Bell Laboratories, USA). The presentation of the stimuli was triggered by the scanner. All stimuli were projected in a circular aperture with a diameter of 15° visual angle on a grey background. In the center of each stimulus a red fixation dot (with a radius of 0.08° visual angle) was presented within a circular aperture (with a radius of 0.4° visual angle), which was the same color as the background. The mean luminance of the whole stimulus was 42.2 cd/m^2^ and did not vary during any of the stimulus presentations. The participants were instructed to focus on the fixation dot at all times and attention to the fixation dot was controlled (see below). In total, five different stimuli were presented: two retinotopic mapping stimuli (polar angle and eccentricity mapping) and three motion stimuli (coherent motion, motion with spatial decorrelations, and motion with temporal decorrelations).

#### Retinotopic mapping stimuli

Retinotopic maps were acquired using a polar angle mapping stimulus and an eccentricity mapping stimulus. The polar angle mapping stimulus was a rotating wedge with a length of 7.5° visual angle. The width of the wedge was 45° circular angle. The wedge made 4 full rotations: twice clockwise and twice counterclockwise. A total of 192 images was acquired during the polar angle mapping. The eccentricity mapping stimulus was an expanding and contracting ring with a width of 1.5° visual angle, which was 1/5th of the maximum eccentricity (7.5° visual angle). Similar to the polar angle mapping, the eccentricity mapping completed 4 cycles: twice as an expanding ring and twice as a contracting ring. During the eccentricity mapping, 180 images were acquired. Both mapping stimuli consisted of a black and white checkerboard pattern, which switched contrast every 125 ms.

#### Coherent motion stimulus

**Figure 2:**
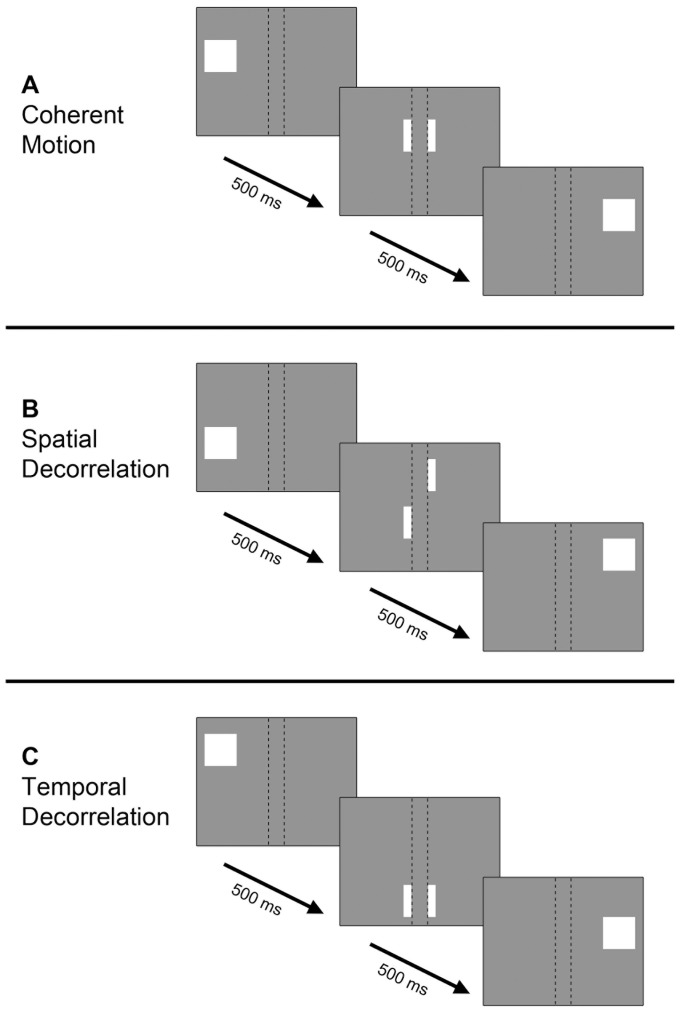
Simplified schematic of motion stimuli. The behavior of a single dot in timeframes of 500 ms is shown. The occluding bars are denoted by dashed lines, which were not visible during the actual experiments. Coherent motion (A): the dot moves in a straight line. Spatial decorrelation (B): dot moves in a straight line until an occluder, where it is randomly repositioned alongside the other end of the occluder. Temporal decorrelation (C): dot moves in a straight line and is randomly repositioned within the stimulus every 500 ms.

The first stimulus was a moving random dot pattern that showed motion at full coherence (figure 2A). The dot pattern was presented within a circular aperture with a radius of 7.5° visual angle. The entire pattern consisted of approximately 2400 square dots with a width and height of 0.38° visual angle, which were randomly distributed within the circular aperture. Most dots partially overlapped other dots. The dots were 50% black and 50% white and moved at a constant speed of 3.4°/s. In addition, the stimulus was partially occluded by 9 thin bars (0.075° visual angle), placed orthogonally to the path of motion. The occluding bars were never wide enough to block-out an individual moving dot completely. When a dot reached the stimulus borders, it was randomly redistributed at the other extremity of the stimulus. A block of moving dots lasted for 15 seconds (10 functional images), which was alternated with a 15 seconds rest block showing static dots. During a motion block, the dot pattern moved in 1 of 4 directions: rightwards, downwards, leftwards or upwards. The thin occluding bars were repositioned every time the direction of motion altered, so that their position was orthogonal to the path of motion. In total, one session consisted of 4 cycles, in which all 4 motion directions were presented. During a session 320 images were acquired (Movie S1).

#### Spatial decorrelation stimulus

The second moving random dot stimulus had the same main characteristics as the coherent motion stimulus described above. However, this stimulus included spatial decorrelations (figure 2B). The path of a moving dot was disrupted after each occluding bar, located at spatially fixed points within the stimulus. When a dot approached an occluding bar, it gradually disappeared. The dot was randomly repositioned along the length of the bar, where it gradually reappeared. The disruption resulted in ten strips of motion between the nine thin occluding bars and the edges of the stimulus aperture (Movie S2). Therefore, the spatial decorrelations confined the path length of coherent motion to the motion path length in between occluders and stimulus apertures.

#### Temporal decorrelation stimulus

The third stimulus was a moving random dot pattern, in which the motion stimulus was temporally decorrelated (figure 2C). All properties of the moving dot pattern were the same as the coherent motion stimulus, except that the dots were randomly and simultaneously redistributed across the entire stimulus every 500 ms (the same duration it took a dot to travel between bars during the spatial decorrelation stimulus). During the stationary period, the dot pattern was also redistributed across the stimulus every 500 ms to control for BOLD signal changes solely caused by the sudden change in contrast of redistributed dots (Movie S3). The path of motion of an individual dot lacked continuity due to disruptions of the stimulus at fixed points in time, while motion remained fully coherent between the dot rescrambling. Thus, the spatial range of coherent motion stretched out over the entire length of the stimulus, while the temporal motion coherence was disrupted every 500 ms.

#### Attention task

During all experiments, an attention task was presented to ascertain that subjects kept their eyes and spatial attention fixed at the center of the stimulus regardless of the motion direction. During the motion stimuli, a white cross was projected every 1000 ms on top of the red fixation dot. During approximately 25% of all 480 cross-projections an attention cue was presented, where the white cross was accompanied by a white arrow pointing in one of four directions: left, right, up or down. The participants were instructed to respond with a button press, using a button box with four buttons, that corresponded to the direction of the presented arrow. The inter-trial interval and arrow-direction were randomized.

### Statistical Analysis

**Figure 3:**
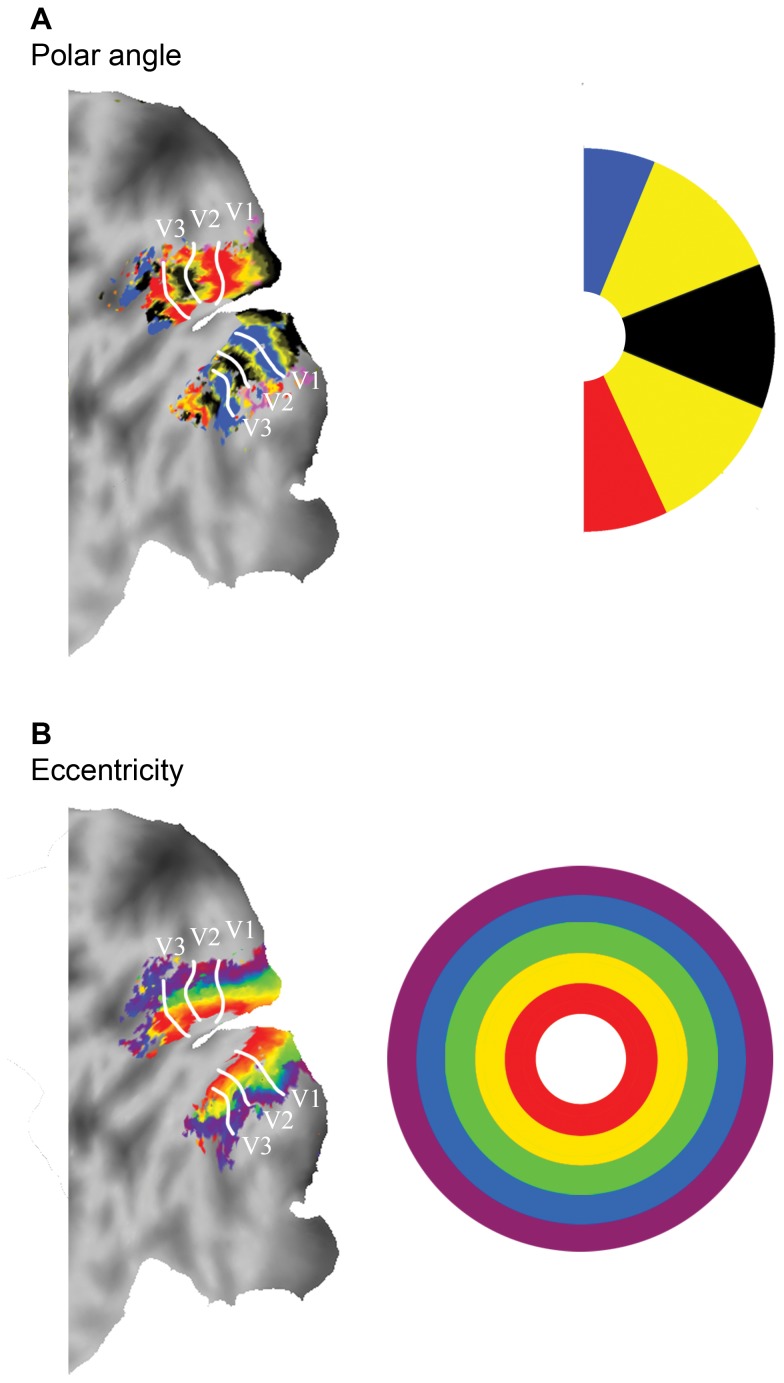
Retinotopic mapping. Results from the polar angle (A) and eccentricity mapping stimuli (B) on a flattened cortical surface representation of the left hemisphere of one subject (JK). The color bars denote the 4 different polar angles (half of the hemifield) and all 5 eccentricities. The separate visual areas are marked by the white lines.

All functional images were spatially preprocessed using SPM8 (http://www.fil.ion.ucl.ac.uk/spm/). The preprocessing entailed the realignment of all scans to the mean scan, slice time correction and coregistration to the anatomical image. The T1 image was corrected for field inhomogeneities by dividing the T1 image by the proton density image as described by Van de Moortele et al. [18]. Afterwards the corrected T1 image was loaded into the Computerized Anatomical Reconstruction and Editing Toolkit (CARET, [19]). The image was resampled to 1 mm isotropic and manually placed into Talairach orientation [20]. By determining gray/white matter intensities, the middle layer of gray matter was estimated and used to reconstruct a surface per hemisphere. Subsequently, the surface reconstruction was inflated and several cuts were applied, among which were cuts along the calcarine fissure and medial wall to obtain a flat map of the corresponding hemisphere. All functional images were mapped onto the surfaces of the left and right hemispheres, using a metric Gaussian mapping algorithm, resulting in a timeserie for every node of the surface. Low frequency noise was removed using multiple regression and a design matrix containing the mean of each image and four cosine functions per experiment, which formed a high-pass filter with a cutoff at 4.2×10^−3^ Hz. For the retinotopic mapping experiments a phase-encoded regressor-matrix was used. The regressor-matrix contained a regressor for every scan during a stimulus cycle and represented the cyclic activation during the presentation of rings (8,000 ms activation every 60,000 ms) and wedges (8,000 ms activation every 64,500 ms) and was convolved with a hemodynamic response function [21]. A correlation coefficient was calculated for every regressor in the regressor-matrix (i.e. every image in a cycle) for every node of the reconstructed surface. The peak correlation of a node determined the eccentricity or polar angle of a node’s receptive field. The eccentricity was interpolated over 5 steps: 1.42° visual angle per eccentricity, which covered the maximum width of the stimulus ring. The polar angle coefficients were interpolated over 8 steps, including 4 cardinal and 4 oblique segments. The visual areas were segmented by drawing borders on the flat representation of the (non-interpolated) polar angle and eccentricity results and contained the striate and extra-striate areas V1, V2, and V3 (figure 3).

For the analysis of the motion stimuli, only 4 of all 8 polar angle steps were used, 45° circular angle each, that covered the horizontal (left and right) and vertical (top and bottom) meridians, corresponding to the four directions that were used during the motion stimuli. The procedure resulted in 60 segments (4 polar angles×5 eccentricities×3 visual areas). The average amount of nodes per segment was m = 115.8 with a standard deviation of σ = 55.3 (Table 1). For each segment the percentage of BOLD signal increase was calculated for each relative motion direction (i.e. centripetal, centrifugal and tangential motion direction). The amplitude of the signal increase was estimated using a linear regression, resulting in a beta-value (β-value) for each segment and motion direction. To test for significant effects, a univariate GLM (general linear model) repeated measures design was adopted for each motion experiment with the following layers: relative motion direction (centrifugal, centripetal, tangential) ×visual area (V1, V2, V3) ×eccentricity (the 5 eccentricity segments). Using Mauchly’s test of sphericity [22] the variables (β-values) in the univariate repeated measures design were tested for violations of the sphericity assumption. When a variable did not pass the sphericity test, the degrees of freedom were adjusted using Greenhouse-Geisser’s epsilon [23].

**Table 1:** Mean number of surface vertices per mapping segment.

	Eccentricities				
	0.40°–1.82°	1.82°–3.24°	3.24°–4.66°	4.66°–6.08°	6.08°–7.50°
Upper visual field	220.3	82.5	62.2	52.8	206.9
Left visual field	177.1	114.6	74.6	62.1	169.8
Lower visual field	149.9	82.6	69.8	52.5	145.1
Right visual field	182.1	108.0	83.2	60.9	158.5

Mean number of surface vertices per polar angle visual field representation, 45° circular angle each, per eccentricity.

To compare the BOLD amplitude of directional biases between stimuli, we calculated the amplitude of the centripetal bias (β-difference between the centripetal and tangential motion directions) and the centrifugal bias (β-difference between the centrifugal and tangential motion directions) for each eccentricity. This was done to control for differences in amplitude of motion responses relative to baseline and, therefore, for the different baseline conditions of the motion stimuli. The differences between stimuli in amplitudes of the biases were then tested for significance for each eccentricity using separate T-tests. A MANOVA test (Wilks’ lambda) was used to test for differences in the performance on the attention task between motion experiments.

## Results

### Coherent Motion Stimulus

**Figure 4:**
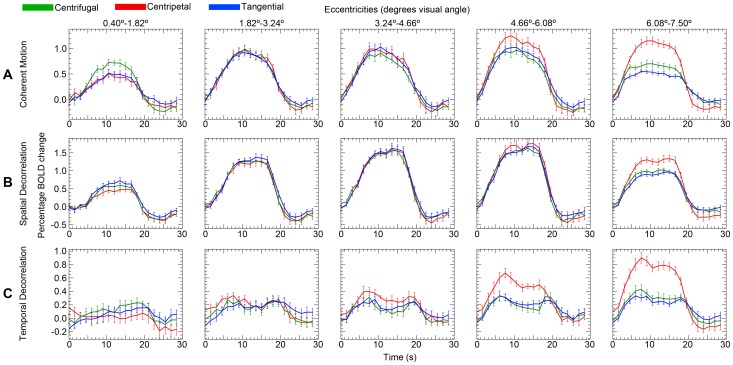
Signal change motion experiments. Percentage of BOLD signal change (mean V1, V2, V3) is plotted over time (s) for all three motion experiments (n = 11). Separate eccentricities are plotted in separate graphs from left to right. The separate lines denote the different motion directions. The error bars denote the standard error of the mean across subjects.

**Figure 5:**
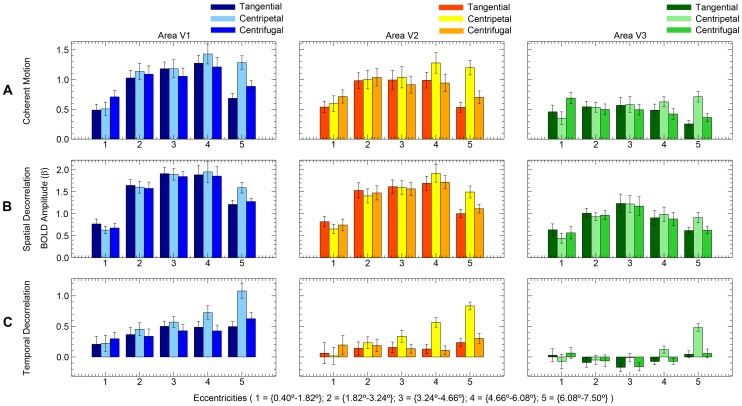
Amplitude motion experiments. The estimated BOLD amplitude (beta) is plotted over the separate eccentricities (n = 11). The results from the separate visual areas are plotted from left to right. The colored bars denote the different motion directions. Error bars denote the standard error of the mean across subjects.

The relative motion direction (i.e. centripetal, centrifugal and tangential motion direction) had a significant effect on the BOLD amplitude during the presentation of coherent motion (F_(2,20)_ = 12.9, p<0.001), indicating the presence of directional motion biases (figure 4A). However, there was no significant interaction between relative motion direction and visual area (F_(4,40)_ = 2.4, p = 0.069). Figure 5A shows the differences in BOLD amplitude among the visual areas and also shows the presence of biased responses in all three visual areas. There was a strong interaction between eccentricity and motion direction (F_(3,32)_ = 32.3, p<0.001). The interaction between eccentricity and motion direction is also visible in figure 4A; a centrifugal bias was mainly observed in the inner eccentricity (0.4°–1.82°), while a centripetal bias was observed in the outer eccentricities (4.66°–7.5°). These results show that we were able to replicate the directional motion biases as reported by Raemaekers et al. [4].

### Spatial Decorrelation Stimulus

The relative motion direction had a significant effect on BOLD amplitude in the spatial decorrelation motion stimulus (F_(2,18)_ = 3.6, p = 0.048). There was no significant interaction between motion direction and visual area (F_(4,36)_ = 1.0, p = 0.400). However, there was a strong interaction between motion direction and eccentricity (F_(8,72)_ = 14.7, p<0.001), which can be attributed to the small centripetal bias at the border of the stimulus (6.08°–7.5° eccentricity). A centrifugal bias was not present at inner eccentricities (figure 4B). In sum, we found a small centripetal bias in the far periphery of the stimulus, whereas we found no biased responses for any motion direction in the remaining parts of the stimulus.

### Temporal Decorrelation Stimulus

The temporal decorrelation stimulus resulted in directional motion biases (figure 4C); the effect of motion direction was significant on the BOLD amplitude (F_(2,20)_ = 21.2, p<0.001). The interaction effect between motion direction and visual area was also significant (F_(4,40)_ = 3.2, p = 0.022), as was the interaction between motion direction and eccentricity (F_(4,38)_ = 22.9, p<0.001). This latter interaction is also displayed in figures 4C and 5C, for a large centripetal bias was measured in the outer eccentricities (3.24°–7.50°) and a small centrifugal bias was present in the inner eccentricity (0.40°–1.82°). These results show that the temporal decorrelation of moving dots did not remove the anisotropy in directional motion responses.

### Comparison between Motion Stimuli

The differences in BOLD amplitude between centripetal and tangential (centripetal bias) and centrifugal and tangential (centrifugal bias) motion directions were compared between stimuli to investigate the effects of the stimulus type on the presence of motion biases using a normalized measure. In the innermost eccentricity (0.40°–1.82°), coherent motion showed a centrifugal bias, which was significantly larger compared to motion with spatial decorrelations (T_(20)_ = 3.606, P = 0.002), but not compared to motion with temporal decorrelations (T_(20)_ = 1.597, P = 0.126). The centrifugal bias difference between motion with temporal and spatial decorrelations, however, was not significant in eccentricity ‘0.40°–1.82°’ (T_(20)_ = 1.873, P = 0.076).

Centripetal biases were measured in the periphery of the motion stimuli. In eccentricity ‘4.66°–6.08°’, the centripetal bias was not significantly larger for coherent motion compared to motion with spatial decorrelations (T_(20)_ = 1.152, P = 0.263). A large centripetal bias was also measured for motion with temporal decorrelations in eccentricity ‘4.66°–6.08°’, which did not differ from coherent motion (T_(20)_ = 1.577, P = 0.130). However, the centripetal bias in eccentricity ‘4.66°–6.08°’ during motion with temporal decorrelations was significantly larger compared to motion with spatial decorrelations (T_(20)_ = 3.288, P = 0.004). Although all motion stimuli displayed a centripetal bias in the outermost eccentricity (6.08°–7.50°), the centripetal bias was significantly larger for coherent motion compared to motion with spatial decorrelations (T_(20)_ = 2.465, P = 0.023), while the centripetal bias did not differ between coherent motion and motion with temporal decorrelations (T_(20)_ = 0.438, P = 0.666). Finally, the centripetal bias in eccentricity ‘6.08°–7.50°’ was significantly larger for motion with temporal decorrelations compared to motion with spatial decorrelations (T_(20)_ = 2.183, P = 0.041). These results show that motion with temporal decorrelations resulted in similar motion biases compared to coherent motion. However, motion with spatial decorrelations only showed centripetal biases in the periphery of the stimulus, which were smaller than the centripetal biases of the other motion stimuli in the same region.

Note that the percentage of BOLD signal change relative to the stationary-dot condition differed substantially between stimuli (figure 4). On average the signal increase during motion with temporal decorrelations was smaller than the other motion experiments. Possibly the rescrambling of the dot positions during the reference condition of the temporal decorrelation experiment may have elevated the baseline activation. In addition, the baseline elevation appeared to differ per visual area (figure 5), while motion biases remained significantly present. There was no dot rescrambling during the reference condition of the other two stimuli.

### Attention Task

Performance data of the attention task was collected from the 3 motion experiments (11 subjects each). Of the 33 sets of psychophysical data 8 sets were excluded due to technical problems with the button box. The remaining 25 sets of attention task data resulted in 71.9% correct button presses (i.e. after a cue the corresponding button was pressed before the next cue was presented). The percentages of missed and incorrect button presses during the attention task were 27.0% and 1.1% respectively. There was no difference in performance on the attention task between experiments (F_(6,40)_ = 0.752, P = 0.611).

## Discussion

### General Discussion

In this study we presented three motion stimuli to investigate two possible types of motion integration that could underlie directional motion anisotropies in retinotopic areas V1, V2 and V3. As in Raemaekers et al. [4], we found directional motion anisotropies for centrifugal and centripetal motion directions during the presentation of coherent motion. The current results only slightly differ with respect to the centripetal bias in lower eccentricities, that was absent during our motion experiments. This may be caused by the current study’s larger stimulus area; no motion was presented in the highest eccentricity in the study of Raemaekers et al. [4]. In contrast to coherent motion, motion with spatial decorrelations showed a centripetal bias only in the outermost eccentricity of the stimulus and no centrifugal bias, whereas motion with temporal decorrelations resulted in motion biases similar to coherent motion.

If directional motion biases depend on an integration of spatial information of aligned motion detectors along the path of motion, then a disruption of motion coherence at fixed points in space will diminish motion biases, while a disruption of motion coherence at fixed points in time will not. On the other hand, if a temporal delay between aligned motion detectors is included in the integration of motion responses, then motion with either temporal or spatial decorrelations will result in a disappearance of directional motion biases. The spatial decorrelation stimulus showed that disrupting the path of moving dots at fixed points in visual space results in a disappearance of motion biases, except for a small centripetal bias in the periphery of the stimulus. However, when motion is disrupted at fixed points in time, directional motion biases will emerge similarly compared to coherent motion.

Although both hypothesized types of motion integration predict a complete disappearance of motion biases during motion with spatial decorrelations, a small centripetal bias in the periphery of the stimulus is observed during motion with spatial decorrelations. A putative explanation for the presence of this peripheral bias may be that a decorrelation at a fixed point in space does not always fully disrupt the activity of a motion detector chain at that point in the visual field. On-off detectors, which motion detectors are thought to pool from, are known to have overlapping receptive fields [24], [25], and receptive field size increases with eccentricity [26], [27]. Motion detectors that pool from cells with overlapping receptive fields can detect spatiotemporal motion coherence across a spatial decorrelation, which would result in a failure to effectively disrupt the motion detector chain. The larger the receptive fields, the more likely it becomes that motion detectors remain unaffected by small spatial decorrelations. This could cause a differential effect for the fovea and the periphery as is observed in the current study. This explanation is supported by the fact that directional biases were completely absent in a previous study [4], when large occluding bars were used instead of decorrelations to interrupt the motion detector chain. Large occluding bars will disrupt the motion detector chain, even for large overlapping receptive fields. Alternatively, the decrease or disappearance of motion biases during motion with spatial decorrelations and orthogonally placed occluders, could be related to the presence of orthogonally oriented (second-order) motion contours [2], [28]. Motion-defined boundaries are clearly present at the spatial decorrelation locations and are orthogonally oriented relative to the direction of motion, which could possibly nullify any radial motion bias. However, such a scenario cannot explain, why specifically centrifugal *or* centripetal motion directions are affected, while second-order motion contours would affect centrifugal and centripetal motion directions to a similar extent. The current results, thus, indicate that it is not the traveled distance of individual dots that causes the motion biases, but rather the spatial length of an activated motion detector chain. Directional motion biases most likely result from a spatial instead of spatiotemporal integration of motion detector activity.

The dependence of directional motion biases on the spatial extent of an activated motion detector chain may be related to motion recruitment [6], [9], [10]. The psychophysical study of Van Doorn et al. [6] showed that motion detection mainly depends on the length of the path of motion and that the number of estimated activated motion units increases with a power of 1.6^th^ of the motion path length. Thus, motion sensitive units are progressively recruited in the direction of motion. In light of current results, motion biases may be a product of motion detector recruitment, which is aborted when a motion detector chain is interrupted. In addition, it has been suggested that the summation of detector information in motion recruitment is linear [8], [29]. The centripetal bias during motion with spatial decorrelations can be the result of a linear spatial integration, given an ineffective disruption of the motion detector chain in the periphery of the stimulus.

The current results indicate that directional motion biases are most likely related to contextual or extra-classical receptive field effects instead of local inhomogeneities in detectors tuned for a particular motion direction. However, the mechanisms behind these extra-classical receptive field effects are still unknown. One possibility is that top-down influences from areas such as MT or MST play a role, as these areas are known to contain mechanisms for global motion perception of translating objects [30]–[32]. Theoretical frameworks of global motion perception have included the integration of local spatial as well as temporal motion information [17], [33], [34]. Additionally, recent studies on global motion perception suggest the presence of an adaptive pooling mechanism, allowing the visual system to switch between motion integration mechanisms, depending on the availability of particular motion information [35], [36]. However, upstream areas related to global motion perception (e.g. MT and MST), have large receptive field sizes [37], [38] and are not believed to be specifically sensitive to differences in spatial and temporal discontinuities, and subsequently provide differential feedback. Alternatively, top-down processes and feedback loops are present within early visual areas as well [39], [40]. For example, extraclassical receptive field effects could be mediated through long-range horizontal connections. Long-range horizontal connections are known to cover large areas of striate and extra-striate cortex and have also been reported to facilitate contour and orientation detection [41], [42]. In addition, it has been suggested that motion and orientation biases share a mutual underlying mechanism [2], [43], [44]. Clifford et al. [2] suggest that directional motion biases arise as a result of blurred temporal integration, resulting in motion streaks. Depending on the orientation of a motion streak, motion biases might emerge, which directly links motion biases to orientation biases. However, motion streaks cannot explain why the current experiment is able to discriminate between centripetal and centrifugal motion biases, since for both motion directions the motion streak would be roughly the same. As the presence or absence of directional biases is dependent on local features of the motion stimulus, we believe anisotropies in long-range horizontal connections or other forms of local connectivity are at least necessary for the emergence of the directional motion biases.

There are a couple of factors that could have confounded the observed findings. Firstly, there is the possibility that the motion stimuli induced different eye movements for different motion directions. Eye movements are known to potentially influence low-level activity within the (extra-) striate cortex [45]. Secondly, covert spatial attention is also known to locally enhance visual responses [46], [47] and motion could induce an attentional drift in the direction of motion or opposite to the direction of motion. However, in a previous study we found that directional motion biases are not related to differences in the fixation position nor the direction of microsaccades during different motion directions, while using similar stimuli as the current study [4]. Furthermore, subjects performed an attention task to keep their eyes and spatial attention fixed on the center of the stimulus. Performance on this task was well above chance (72% correct) and did not differ between the motion experiments. Another possible confounding factor is the usage of a different baseline condition for the temporal decorrelation stimulus with respect to the other motion stimuli. As is reported in the results section, the BOLD-response to motion with temporal decorrelations were considerably more noisy than the BOLD-responses of the other motion stimuli. Furthermore, the transient responses to the repetitive redistribution of dots every 500 ms, might have altered neuronal responses by means of adaption to contrast or changes to motion-after effects. However, we did find the same pattern of directional motion biases for motion with temporal decorrelations and coherent motion. One would expect that, if repetitive transient responses had an effect on directional motion biases, the pattern of motion anisotropy would differ from coherent motion. In addition, the redistribution of dots will briefly activate motion sensitive neurons with direction preferences other than the direction of the stimulus motion. This could possibly lead to a brief bistable percept or other effects, such as reverse-phi like phenomena [48], [49]. However, the redistribution of dots was random and, thus, would equally stimulate motion detectors with different direction preferences. Furthermore, dot redistribution with equal contrast change was also present during baseline condition, which could lead to the exact same effects. It is, therefore, unlikely that the redistribution of the dots has confounded the observed BOLD signal changes.

Future research should address the nature of these directional biases in light of functional and evolutionary benefits. It would be interesting to investigate, as to whether the absence or presence of directional motion biases can be related to certain perceptual qualities, e.g. the saliency of coherent motion presented on a certain background [50], [51]. The role of the different visual areas on directional motion biases should also be of future interest. For coherent motion there was no interaction between motion direction and visual areas, while during motion with temporal decorrelations there was a significant interaction. This finding might represent important clues on the different role of striate and extra-striate areas on motion biases in terms of feedforward- and feedback loops. Further attention should also be devoted to the presence of biased responses near the edges of a motion stimulus. Biases seem more pronounced near the edges of the stimulus in combination with a particular motion direction. This may indicate a relationship with the novelty of visual input. Neuronal output that is influenced by the novelty of visual input might be related to models on predictive coding [52], [53].

### Conclusions

The current study provides evidence that directional motion biases are related to a (linear) spatial integration as opposed to spatiotemporal integration of motion information parallel to the path of motion. Motion biases occur when multiple aligned motion detectors parallel to the path of motion are simultaneously activated. When the length of the path of coherent motion is shortened, motion biases decrease or even disappear.

## Supporting Information

Movie S1 **Coherent motion.** The coherent motion stimulus displays coherent motion, moving in all 4 directions and alternated with a stationary dot rest period. The duration of motion and rest blocks are shorter in this movie (3s), than during the actual experiments (15s).
(AVI)

Movie S2 **Spatial decorrelation stimulus.** The spatial decorrelation stimulus displays motion that is decorrelated at fixed points in space, moving in all 4 directions and alternated with a stationary dot rest period. The duration of motion and rest blocks are shorter in this movie (3s), than during the actual experiments (15s).
(AVI)

Movie S3 **Temporal decorrelation stimulus.** The temporal decorrelation stimulus displays motion that is decorrelated at fixed points in time, moving in all 4 directions and alternated with a stationary dot rest period that is also decorrelated at fixed points in time. The duration of motion and rest blocks are shorter in this movie (3s), than during the actual experiments (15s).
(AVI)
